# Considerations and analysis of the implementation of oncogeriatrics in Chile and its importance: Review of current literature

**DOI:** 10.3389/fragi.2023.1141792

**Published:** 2023-03-23

**Authors:** Macarena Honorato, Oscar Calderón, Verónica Rojas, Gerardo Fasce, Constanza Bartolotti, Christian Caglevic

**Affiliations:** ^1^ Geriatrics Department, Clínica Alemana de Santiago, Santiago, Chile; ^2^ Geriatrics, Complejo Asistencial Dr Sótero del Río, Santiago, Chile; ^3^ Geriatrics Service, Department of Medicine, Hospital Clínico Universidad de Chile, Santiago, Chile; ^4^ Geriatrics Service, Department of Medicine, Clínica Las Condes, Santiago, Chile; ^5^ Geriatrics, Internal Medicine Department, Universidad de la Frontera, Temuco, Chile; ^6^ Geriatrics, Centro Comunitario Kiműnche, Temuco, Chile; ^7^ Cancer Research Department, Instituto Oncológico Fundación Arturo López Pérez, Santiago, Chile

**Keywords:** oncogeriatric assessment, geriatics, elderly, cancer, Chile

## Abstract

The Chilean census of 2017 reported that 11.4% of the local population are 65 years or older, and according to the National Institute of Statistics (INE) the current expectancy of life in Chile is 76 years for men and 81 years for women respectively. Cancer in Chile is a major public health problem. Aging is a significant risk factor for cancer development which added to the improved life expectancy, it increases the incidence of cancer. In 2040, new cancer cases will increase from 19.3 to 30.2 million worldwide. Older people are a heterogeneous group requiring specialized and individualized management. Chronological age does not necessarily correlate with physiological age. More than half of the geriatric patients with cancer have at least one comorbidity which is relevant when defining a cancer treatment. Likewise, polypharmacy is frequent and is an important issue to consider in people with cancer due to the risk associated with drug interactions. Oncogeriatric assessment consists of a comprehensive multidimensional evaluation, including functional and biopsychosocial issues, addressing aspects of the neoplastic disease such as the risk of toxicities due to systemic therapy and life expectancy. This tool has proven to be helpful in the diagnosis of conditions that are not evident in a routine oncological evaluation, such as geriatric syndromes, frailty, functional dependence, and cognitive impairment among others, which have an impact when deciding on therapy, predicting risks of treatment toxicity and mortality. In this article we aim to describe the current situation of Oncogeriatrics and to provide epidemiological information about cancer in the elderly population in Chile attempting to highlight the importance of the Oncogeriatrics units, within cancer departments, for a better decision taking in the elderly cancer patient.

## Introduction

Aging is a well-recognized risk factor in carcinogenesis (Hoffe and Balducci, 2012), directly involved in the expected increase of new cancer cases from 19 to more than 30 million by 2040 (International Agency for Research on Cancer, 2023).

According to official information, 11.4% of the Chileans are 65 years or older, with an expectancy of life of 76 years for men and 81 for women (Instituto Nacional de Estadísticas de Chile INE, 2023; International Agency for Research on Cancer, 2023) The elderly population in Chile accounts for 3,348,010 inhabitants, 1,861,067 females, and 1,468,943 males (Instituto Nacional de Estadísticas, 2023), 90% of them are part of the Chilean public system of health (FONASA, 2023), and the rest are treated in the Chilean private system of health (ISAPRES).

Currently, cancer is the main cause of death in Chile. Unfortunately, local statistics were not initially designed thinking of the definition of the geriatric population, therefore the reported incidence of cancer for the group of 50–69 years is unique and does not allow to have confident numbers concerning the 60–69 years subgroup. Also, there is a lack of an integrated national register of tumors, a situation that underestimates the real dimension of the cancer patient among the geriatric population.

Among the 70 years and older Chilean population, the reported incidence rate and percentage for this group of age for the main type of tumors are as follows: prostate cancer (345.3/100,000 male, 22.9%), gastric cancer (247.7/100,000 inhabitants, 16.4%), and lung cancer (193.1/100,000 inhabitants, 12.8%) for the male population; and gastric cancer (96/100,000 inhabitants, 10.9%), lung cancer (94.2/100,000 inhabitants, 10.7%), and breast cancer (83.9/100,000 women, 9.5%) for the female population (de Mortalidad, 2023). Table 1 and Table 2 show the three main types of malignancies among 70 years and older population by sex, in Chilean population during the period 2009–2018.

**TABLE 1:** Main types of malignant tumors excluding non-melanoma skin cancer, total number of cases, percentage representing among 70 years and older population, mortality rate, in Chilean male population during the period 2009–2018.

Cancer type	Total number of cases	% That represents among 70 years and older male- Chilean-population	Mortality rate (per-1,00,000)
Prostate cancer	16,976	22.9%	345 per-100,000 men
Gastric cancer	12,179	16.4%	247 per-100,000 inhabitatants
Lung cancer	9,492	12.8%	193 per-100,000 inhabitants

Data source: “MINSAL, Informe de Vigilancia Epidemiológica de Cáncer, Análisis de Mortalidad, Década 2009–2018, Chile 2020, Departamento de Epidemiología” Ref 11.

**TABLE 2:** Main types of malignant tumors excluding non-melanoma skin cancer, total number of cases, percentage representing among 70 years and older population, mortality rate, in Chilean female population during the period 2009–2018.

Cancer type	Total number of cases	% That represents among 70 years and older female- Chilean-population	Mortality rate (per-100,000)
Gastric cancer	7,002	10.9%	96 per-100,000 inhabitants
Lung cancer	8,867	10.7%	94 per-100,000 inhabitants
Breast Cancer	6,120	9.5%	83 per-100,000 women

Data source: “MINSAL, Informe de Vigilancia Epidemiológica de Cáncer, Análisis de Mortalidad, Década 2009–2018, Chile 2020, Departamento de Epidemiología” Ref 11.

Currently, in Chile as in other developing countries, oncogeriatrics is gaining impact little by little. It is being promoted by trained specialists, giving guidelines and tools to geriatricians, medical oncologists, hematologists, radiotherapists, and palliative care physicians to achieve quality care and individualized therapy for geriatric patients with cancer.

Cellular senescense is a process that leads to a permanent cell arrest, avoiding cell proliferation, and when the different mechanisms that are involved in this process are functioning properly, the creation of cancer cells should be controlled. Non-etheless, there are several risk factors, including failures within these control pathways that facilitate the creation of tumor cells, therefore older age becomes a greater risk for developing cancer (Aunan et al., 2017).

Due to efficient health policies in Chile, a significant reduction in the child mortality rate occurred in recent past decades, associated with better control and management of chronic diseases in adults. This equation resulted in a significant increase in the geriatric population.

These results place Chile, along with Cuba and Uruguay within the countries of America that by 2025 will have approximately 20% of the elderly population. This not only means a great achievement in the health policies of these countries but at the same time, a great challenge for providing an adequate standard of life and health to this group, optimizing the management of chronic pathologies, including in this concept the multidisciplinary management of cancer in a more frailty population, often affected by limited socioeconomic resources and access to optimal healthcare (Ortega-Gonzalez, 2018).

In Chile, awareness has been raised about the importance of the comprehensive management of the elderly patient with cancer and the need to expand knowledge about this topic among general practitioners, family physicians, internists, and surgeons, but also in all the health team (MINSAL, 2023). In this country, there is an increase in the cancer rate in part due to the aging of the population. Despite that many cancer registries in Latin America are in process of continuous improvement, there is no exact data on the real number of older people with cancer, often due to insufficient notification, lack of diagnostic verification, or simply limited access of patients to specialized health centers (Rolfo et al., 2016).

## The importance of oncogeriatrics for the cancer teams

Cancer treatments include but are not limited to surgery, systemic treatments (cytotoxic chemotherapy, molecular therapies, biological therapies, hormone therapy, immunotherapy), and radiation therapy. Decision-making on how to properly treat a patient is based on multidisciplinary decisions supported by scientific information from clinical studies. Unfortunately, in clinical studies the elderly population tends to be underrepresented, assuming equivocally that the results of these trials always represent this patient´s population (Denson and Mahipal, 2014). In the decision-making process on cancer treatments, toxicities and potential sequelae must be considered (Nurgali et al., 2018). Due to new drugs and radiotherapy development, rational and precision surgery, and also an earlier diagnosis of cancer, the older people who will survive cancer in the coming decades will increase strongly, however, the effects of these treatments will have repercussions for the survivors with a potential affectation on their quality of life (Shahrokni et al., 2016). Therefore, it is necessary to consider that clinical studies should be adapted for the geriatric population, adjusting their inclusion and exclusion criteria and the objectives that are relevant for this group of age (Whelehan, Lynch, Treacy, Gleeson, Oates, O'Donovan). Many times, the decision-making of cancer treatments is mostly based on the medical oncologist’s opinion assuming that are properly trained for treating older patients with cancer (Lichtman, 2019).

Regardless of the training of cancer specialists, treatment of the elderly is highly complex and requires a vision and assessment by a geriatrician with knowledge of cancer to obtain proper information about the functional and psychological capacities of the patient, helping for therapeutic decision-making by the cancer medical team. The geriatrician dedicated to evaluating and supporting cancer patients, known today as an oncogeriatrician, uses different tools and instruments to perform a comprehensive geriatric report, subdividing patients according to a greater or minor risk of toxicities to therapies and according to the objective expectations of survival with or without systemic treatment, allowing a rational take of decisions (Mohile et al., 2013).

## Characteristics of elderly cancer patients

Older people are a group with special characteristics, very different when compared to the younger population. Therefore, it is important to establish some concepts to clarify the differences between geriatrics and non-geriatric adult patients. Briefly, we will explain physiological changes, and geriatric syndromes which include but are not limited to frailty, polypharmacy, and its consequences, fall syndrome, and malnutrition in the elderly and their relationship with cancer treatments.

There are important physiological changes in the aging process. Knowing the heterogeneity of physiological changes in pharmacokinetics, pharmacodynamics, tolerance in different tissues, and how this influences carcinogenesis is essential for understanding the link between cancer and its treatment in older people. Vulnerabilities assessment, the presence or absence of frailty, and comorbidities are associated with life expectancy (Williams et al., 2016; Balducci and Extermann, 2000).

The objectives in the management of elderly patients with cancer should be individualized according to the context of each person as an individual with some common goals, such as relieving symptoms and complications cancer-related, preventing and reducing treatment-related toxicities, improving tolerance to therapy, improving communication between patients and health personnel, reducing the emotional burden between patients and caregivers, and optimizing the care of survivors.

Within geriatric syndromes, some of them are associated with worse results in some cancer treatments. Frailty is defined as a cyclical, complex, and multidimensional state of reduction of the physiological reserve, resulting in a lower capacity for resilience, adaptation, and increased vulnerability to stressors. In the general population, its prevalence varies between 10% and 20% among people older than 65 years (Collard et al., 2012) and reaches up to 50% in those older than 85 years (Song et al., 2010).

The prevalence of frailty reported among cancer patients ranges from 6% to 86%, with a median of 42% (Handforth et al., 2015). Frailty is associated with a worse survival rate with an HR 2.67 (95% CI 1.11–6.83, p0.029) according to the Linda Fried Frailty phenotype, increasing to an HR 3.39 (95% CI 1.82–6.29, *p* < 0.001) when it is diagnosed by a comprehensive geriatric assessment (Kristjansson et al., 2010). It is also associated with a higher incidence of colorectal postsurgical complications with an OR 4.083 (95% CI 1.4–11.6, p0.006) (Tan et al., 2012) and in gastric surgery with an increase in systemic complications, OR 6.06 (95% CI 1.78–20.9, p0.004) (Lu et al., 2017).

Reported frailty among the Chilean elderly population, based on analysis from the Chilean National Health System, accounts for 10.9% (7.7% for males and 14.1% for females respectively). Depending on the associated morbidity reported frailty was 0% when no comorbidity exists, 6.2% when only one comorbidity is present, but it raises to 64% when three or more comorbidities are present. 32.6% of cancer patients are frailed according to this report (Troncoso-Pantoja et al., 2020).

One of the major problems in geriatrics is polypharmacy (Whitman et al., 2018). There is no consensus for a clear definition for this term (Masnoon et al., 2017), then we could assume that the concomitant use of two or more drugs could be included within this definition. Some authors such as Turner consider polypharmacy when a patient uses five or more different drugs (Turner et al., 2014). Polypharmacy has been associated with a greater probability of interrupting scheduled surgery (Parks et al., 2015), an increase in almost double the risk of post-surgical complications (de Glas et al., 2013), a greater need for hospitalizations (Badgwell et al., 2013), and an increase of six times more grade three or greater chemotoxicities (Hamaker et al., 2014). Polypharmacy is also responsible for producing greater functional deterioration, a higher incidence of delirium (Şenel et al., 2017), and an almost 10-fold increase in the 30-day mortality days in selected populations (Elliot et al., 2014).

Another important geriatric syndrome to be considered and prevented is the “fall syndrome”. The current presence or a previous history of cancer increases the risk of falls by 15%–20% (Mohile et al., 2011). It is essential to ask about the antecedent of falls during the last 6 months, as well as the limitations in activities of daily living, cancer-related fatigue, to assess walking and balance disorders, to request vitamin D plasmatic levels, to review medications in current use, to correct visual disturbances and to carry out close interdisciplinary management with kinesiology and occupational therapy, encouraging and prescribing physical activities with exercises to improve strength and balance.

Malnutrition is a predictor of mortality and morbidity, being related to up to 20% of cancer-related deaths and affecting more than a third of cancer patients (Soubeyran et al., 2012). It is also related to a higher toxicity rate of cancer therapies (Aaldriks et al., 2013), lower response rate, poor quality of life, deterioration of functional status, and prolongation of hospital stay. These results must be considered to develop research strategies and assessments by nutritionists to allow a safer cancer treatment or to inform the oncological team if the patient should not undergo treatment. The presence of mood disorders, pain, taste disturbances, and nausea or vomiting should be also assessed. It is essential to determine access to food and activate the social network, implement, and access nutritional supplements, break down diet myths, and emphasize the importance of protein intake in the diet of the elderly globally.

## Oncogeriatric evaluation models

International recommendations suggest an oncogeriatric assessment for all cancer patients over 65 years old (Hurria et al., 2014; Wildiers et al., 2014; Gironés Sarrió et al., 2018; Loh et al., 2018; Mohile et al., 2018; Molina-Garrido MJ et al., 2019), however, globally there is a lack of geriatricians to achieve this need.

In Spain, after the First National Board of Multidisciplinary Oncogeriatrics Work in 2011, and considering international recommendations as a reference, three ways of evaluating older people with cancer were recommended, depending on the availability and resources of each center (Antonio et al., 2012).

Firstly, the integrated model is considered the gold standard, where a multidisciplinary assessment is carried out involving geriatricians, medical oncologists, radiation oncologists, palliative caregivers, nutritionists, and social workers among other professionals, obtaining a comprehensive diagnosis for making treatment decisions in agreement with the patient. The second form of assessment is the collaborative model which consists of the geriatrician’s support to a cancer specialist in the diagnosis and decision-making process, through consultation. The geriatrician performs the oncogeriatric assessment and provides recommendations. In those health centers that do not have geriatricians, the screening model is carried out, focused on the diagnosis of frailty by the oncologist, through an abbreviated comprehensive geriatric evaluation, to recommend the best type of treatment for the patient considering the chances to achieve better results with minor risk of toxicities.

**FIGURE 1:**
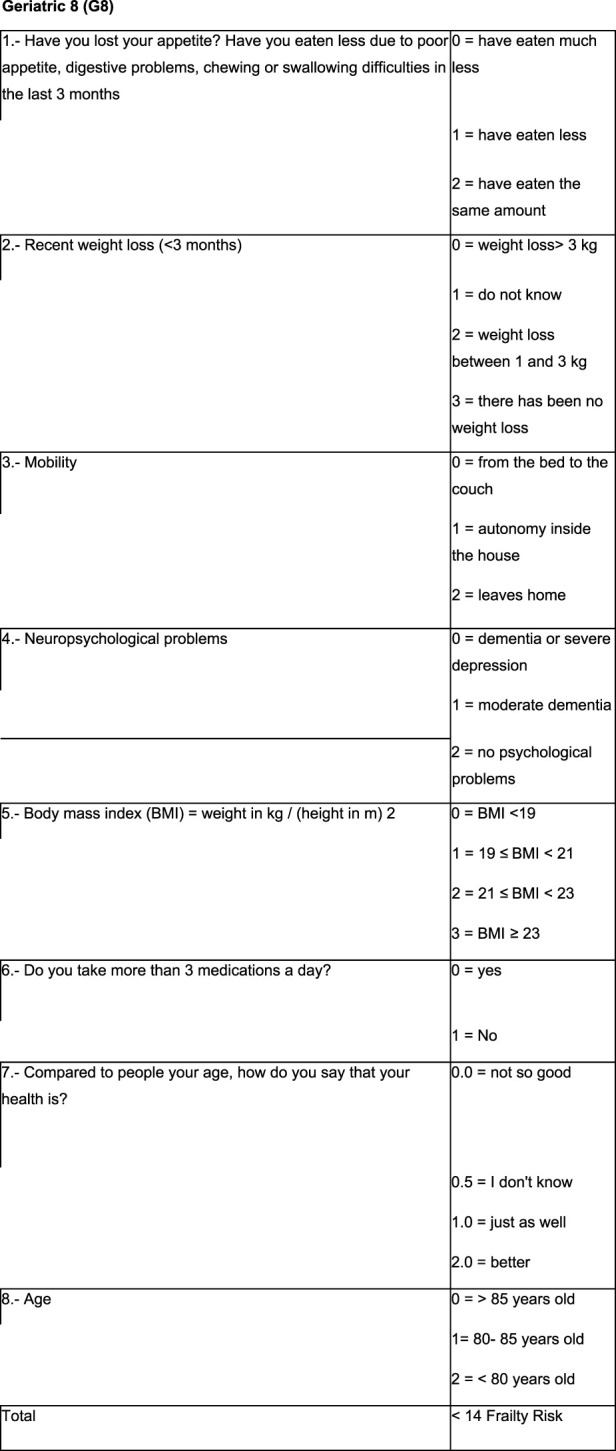
G8 Geriatric assessment questionnaire.

Given the time that is needed for an oncogeriatric evaluation, shortened screening tools have been developed to detect which patients may require an extended geriatric evaluation such as the Geriatric 8 (G8), which consists of eight questions involving some geriatric domains which have shown to be more sensitive in detecting vulnerable older people (van Walree et al., 2019; Figure 1).

A recent official Chilean report demonstrated that the first cause of death in this country is cancer and no longer cardiovascular and circulatory diseases (Ministerio de Salud, 2009-2018), concluding also that the Potential Years of Life Lost are higher among adults from 65 to 69 years when compared with the rest of the population.

To ensure strategies looking for increasing the number of geriatric patients with cancer that may undergo a global assessment, in 2019 the first Oncogeriatrics Update Workshop was held in Chile with the support of the Chilean Geriatrics and Gerontology Society, becoming this meeting the first step of oncogeriatrics in Chile.

Unfortunately, only a few months later, the world started to be badly attacked by the SARS-COV-2 pandemic avoiding wider dissemination of this specialty in Chile.

A report from the United Kingdom showed that patients older than 60 years old with active COVID-19 infection have a significantly higher chance to die of cancer when compared with the younger population (Russell et al., 2021). As in the rest of the world, in Chile, the pandemic has delayed the diagnosis and treatment of cancer patients resulting in an excess of cancer-related deaths (Ward et al., 2021).

Currently, in Chile there are only 178 geriatricians recognized by the Superintendency of Health, most of them are practicing in the Metropolitan Region, which includes nearly half of the country’s population (Superintendencia de Salud de Chile, 2023). This deficit of specialists makes it impossible to follow the international recommendations, nevertheless, some health centers in Chile are already practicing oncogeriatrics. Table 3 shows the institutions that treat cancer patients in Chile and their correlation with the presence or not of an Oncogeriatrics unit or support in this field.

**TABLE 3:** Hospitals and private centers that provide Cancer treatment with or without oncogeriatric units in Chile.

Institution	City	Oncogeriatrics
Hospital Juan Noé	ARICA	no
Hospital Ernesto Torres Galdamez	IQUIQUE	no
Hospital Regional	ANTOFAGASTA	yes
Hospital San Juan de Dios	LA SERENA	no
Hospital Gustavo Fricke	VIÑA DEL MAR	yes
Hospital Carlos van Buren	VALPARAISO	No
Instituto Nacional del Cancer (INC.)	SANTIAGO	Yes
Hospital Salvador	SANTIAGO	yes
Hospital San Juan de Dios	SANTIAGO	yes
Hospital San Borja	SANTIAGO	No
Hospital Sótero del Rio	SANTIAGO	Yes
Hospital Barros Luco	SANTIAGO	no
Hospital Félix Bulnes	SANTIAGO	No
Hospital de Rancagua	O´HIGGINS	No
Hospital de Talca	MAULE	Yes
Hospital las Higueras	TALCAHUANO	yes
Hospital V Rios Ruiz	BIOBIO	No
Hospital G. Benavente	CONCEPCION	No
Hospital Herminda Martin	ÑUBLE	No
Hospital de Temuco	ARAUCANIA SUR	yes
Hospital Base Valdivia	VALDIVIA	No
Hospital de Osorno	OSORNO	No
Hospital de Puerto Montt	RELONCAVÍ	Yes
Hospital de Coyaique	AYSEN	Yes
Hospital de Punta Arenas	MAGALLANES	No
Clinica Alemana	SANTIAGO	Yes
Clínica Bupa Santiago	SANTIAGO	Yes
Clinica Dávila	SANTIAGO	No
Clínica Indisa	SANTIAGO	No
Clínica las Conde	SANTIAGO	Yes
Clinica Redsalud	SANTIAGO	no
Clínica Santa María	SANTIAGO	no
Clínica U Andes	SANTIAGO	no
Fundacion Arturo Lopez Perez	SANTIAGO	yes
Hospital Clínico Universidad de Chile	SANTIAGO	yes
Universidad Católica Christus	SANTIAGO	no
Clínica MEDS	SANTIAGO	no

Within all the institutions, public or private, that provide cancer care, half of them located at Santiago (Metropolitan area) and only half of them have stablished Oncogeriatrics units. Outside the Metropolitan area only 40% of the that provide cancer treatment have oncogeriatrics support.

## Conclusion and final remarks

The increase in the number of elderly people with cancer in Chile is a recognized fact and we must be prepared to face and carry out a multidimensional diagnosis and offer them quality care and appropriate and individualized treatment.

Older people are *per se* a heterogeneous population. Despite the high incidence of cancer among this population, it has historically been an underrepresented group in clinical trials, and we have less data to define a beneficial therapy that implies less toxicity, better tolerance, and quality of life. There are special conditions such as geriatric syndromes that have been associated with undesirable events treatment-related that can be prevented and recognized in a comprehensive geriatric assessment allowing its early management to avoid a worsening in quality of life when possible.

Beyond the challenges that the elderly represent and its relationship with higher chances to develop cancer, there is a lack of trustable information that allows correct comparisons between different Latin American countries to estimate the real burden of cancer among the geriatric population of this part of the world. Currently, Latin America and the Caribbean have 1.5 million new cases per year, but it is expected that by 2040 this number will be increased to 2.4 million new cases per year, and most of these patients will be 65 years and older (Piñeros et al., 2022). In the region, the most frequent types of cancer are prostate (15%), breast (14%), colorectal (9%), lung (7%), and gastric cancer (5%). Lung tumor is still the leading cause of mortality cancer-related. In 2020, the Incidence Age Standardized Rate (ASR) for Latin America and the Caribbean, and Chile was 186.5 and 180.9/100,000 inhabitants, and the Mortality ASR was 86.6 and 87.4/100,000 inhabitants respectively. Despite the relevance of the need for oncogeriatric services in Latin America, there are only three countries with representatives of the International Society of Geriatric Oncology: Mexico, Brazil, and Chile (Verduzco-Aguirre et al., 2020).

Today in Chile there are a dozen of geriatricians with some degree of training in oncogeriatrics but there is not a recognized oncogeriatric unit yet. Half of these professionals work, completely or partially, with an oncological group. 50% of them work in the private system of health, and the rest of them support cancer patients without being integrated into a cancer team. Due to the increase in the elderly population in Chile and the increase in cancer rate among this group, it is essential to promote the development of oncogeriatrics and to create units of this specialty in the country. Spain has created oncogeriatrics units, which will serve as an example to develop similar units in Chile (Martínez-Peromingo et al., 2018), nevertheless, only 14% of the oncological departments account for an oncogeriatrician (Gironés et al., 2018). In the United States, where 50% of cancer patients are 65 years or older, the need to integrate practice nurses into ambulatory care shows to be an effective tool to support oncogeriatric teams (Overcash, 2015).

Considering that in Chile currently there are 25 public oncology services for adults we aim that it should be at least one oncogeriatrician per service.

Interdisciplinary collaborative work is required for subsequent updates and for the creation of local guidelines looking to support the different specialists who increasingly treat more elderly people with cancer in Chile. In addition, we must work to develop an adequate follow-up plan facing early possible difficulties as a result of the treatment complications and the early detection of other oncological pathologies in cancer survivors.

Chile is a country that is having a relevant increase of the elderly population. As mentioned before, currently, cancer is the leading cause of death in the Chilean-adult population including those patients that are involved in the concept of “geriatric age”. Few decades ago, in Chile, the development of oncological treatments was not a priority within the health public policies, however, following the modernization and economic development of the country, cancer treatments, little by little, started to become a need. Despite that the number of specialists in cancer field is still low in Chile, the number of surgical, radiation, and medical oncologists has been increasing during the last decade providing solutions to many regions and cities that had a complete lack of them.

Only by 1997, the first Geriatricians with academic degree were recognized in Chile. By 2023 the number of recognized geriatricians is still low reaching a total of 178 for the whole country. Despite that cancer is currently the leading cause of mortality in Chile, treating adults in geriatrics age offers many times a more complex hazard for the risk of toxicities that are related to treatments that include surgeries, radiation treatments, chemotherapies, immunotherapies, hormonotherapy, biological drugs among others. During the decision-making process, when treating a cancer patient, it is mandatory to consider the wishes of the patient, the expectancy of life in relation with age, cancer and its staging, including also the baseline situation of the patient and comorbidities, the risks of the treatments that can be offered and the potential outcomes of those treatment individualized in a single patient. Clinical trials with oncological drugs and cancer treatments, most of the times, do not consider specific answers for the elderly patient subgroup, and the reported outcomes involve all the adult population from 18 or 21 years-old according to local regulations. Today, all the most recognized cancer centers worldwide perform geriatric assessments for elderly-cancer patients in order to provide a precision tool that helps to define whether a patient should undergo a specific treatment or not and the risks that are involved in. In Chile, there is a lack of geriatricians but also of Oncogeriatrics support. Cancer units have been increasing along all the country and every day more patients have the chance to be treated for their malignancies. Today, the cancer epidemiology includes that, in Chile, more and more elderly patients have malignancies and need a more specific assessment. Recently, in 2010 a small group of geriatricians started to assess cancer patients supporting oncological teams in a better decision-making. By 2023 there is just one Geriatrician and Medical Oncologist doctor in Chile with both academical degrees, but approximately 25 geriatricians that support cancer teams in different hospitals of Chile. Beyond this, only half of the institutions that provide cancer treatments in the Chilean Metropolitan Area, that involves a little less than half of the total population of the country, account with a geriatrician with expertise in cancer. Even poorer is the situation farther away, where geriatricians’ resources that support cancer units are lower than 40% as a total considering all the hospitals that offer cancer treatment within specialized units.

This situation must aware the importance of considering the creation of Oncogeriatrics units in all the hospital that provide a comprehensive cancer treatment in Chile, and to stimulate the formation of more geriatricians and oncogeriatrician.
